# Anticipation and the control of voluntary action

**DOI:** 10.3389/fpsyg.2013.00341

**Published:** 2013-06-14

**Authors:** Dorit Wenke, Rico Fischer

**Affiliations:** ^1^Department of Psychology, Humboldt-Universität zu BerlinBerlin, Germany; ^2^Department of Psychology, Technische Universität DresdenDresden, Germany

A major hallmark in the adaptive control of voluntary action is the ability to anticipate short and long term future events. Anticipation in its various forms is an important prerequisite for cognitive abilities such as planning, reasoning and the pursuit of both immediate goals and long-term goals (e.g., to invest in pension funds) that sometimes stand in opposition to immediate desires and needs. Therefore, it is not surprising that diverse and rather independent research lines have evolved, all somehow targeting various anticipatory capacities that are involved in the control of voluntary action.

One line of research focuses on anticipating action effects. For example, ideomotor theory assumes that actions are selected and activated by the mere anticipation of the sensory experience they produce (James, 1890/1950). Similarly, prediction of the incentive value of action outcomes has been proposed to drive goal-directed instrumental behavior (Balleine and Dickinson, 1998). Furthermore, the degree of match between intended, anticipated and actual action effects seems to be a major determinant of motor programming and online corrections (Prablanc and Martin, 1992), motor learning (Wolpert et al., 2011), and the subjective sense of causing and controlling actions and their effects (the Sense of Agency; Frith et al., 2000). However, the role of anticipation in the control of voluntary action goes beyond the anticipation of action effects. For instance, pre-cues and alerting signals are used for preparing what to do (Meiran, 1996), when to act or expect an event, (Callejas et al., 2004) and for anticipating conflict (Correa et al., 2009). Similarly, learning of statistical contingencies leads to prediction of context-specific executive control requirements (Crump et al., 2006).

The aim of the present Research Topic has been to provide a platform that offers the possibility of cross-fertilization and enhanced visibility among to date rather segregated research lines concerning the role of anticipation in the control of voluntary action.

Many contributions address the role of anticipating action effects in controlling and understanding actions. Some deal with the role of anticipated value of action outcomes: Watson et al. (2012) provide a review on maladaptive drug seeking behavior from a learning theory perspective. Pezzulo et al. (2013) propose a model in which a single mixed controller balances habitual choice based on cached action values, and mental simulations of action outcomes that underlie goal directed behavior, depending on the usefulness of obtaining new information. Scherbaum et al. (2012) propose a model of temporal discounting—the tendency to choose smaller rewards delivered sooner instead of larger rewards delivered later—that focuses on response threshold and time framing as two factors determining choice behavior in inter-temporal choice.

Further contributions address the role of effect anticipation and feedback evaluation in the control and experience of action: Schilling and Cruse (2012) propose a predictive body model for planning robots' actions. Wang et al. (2012) present data showing that distorted visual movement feedback tends to affect action evaluation more strongly in old than in young adults. Haering and Kiesel (2012) demonstrate that prior causal beliefs influence intentional binding, a temporal illusion often seen as an indirect measure of sense of agency. Hommel and Keizer (2012) show that that object files can contain evaluative information regarding the match (success) viz. mismatch (failure) between predicted and experienced events. Poehlman et al. (2012) argue that supramodal integration through conscious states is primarily related to the skeletal muscle output system where anticipatory processes play a central role.

Thinnes-Elker et al. (2012) discuss different concepts of intention with respect to their implications for brain-machine-interfaces that “decode” brain activity for controlling artificial effectors. Because anticipating the consequences of one's own and others actions is an important aspect of social interactions and sport settings, Weigelt and Memmert (2012) investigated how the implicit processing of the stimulus layout in natural scenes affects the goal-side selection in soccer penalty shooting.

Predictive mechanisms are also involved in our ability to understand other people's actions, and even infants tend to interpret various action components with respect to action goals. In this line, Daum et al. (2012) demonstrate a dissociation between two measures often used to investigate expectations about goal-directed actions in infants, namely post-hoc looking times and predictive gaze. Henrichs et al. (2012) report evidence for an impact of goal salience on infants' goal anticipations of observed reaching actions, as measured by predictive gaze.

Another group of contributions focusses on the role of implicit or explicit cues that are utilized by the cognitive system to adjust cognitive control: Wendt et al. (2012) show that cue-based task preparation during task-switching is modulated by the validity of preceding trial task-cues. Strack et al. (2013) investigated cue-induced preparation, aiming at disentangling anticipatory control adjustments and prevention of upcoming conflict via task recoding. King et al. (2012) applied a model-based analysis and argue that context-specific proportion congruence effects may be accounted for by a prediction error-triggered shift in the decision criterion. Bugg and Crump (2012) provide a review on list-wide, item-specific and context-specific proportion congruence effects. Reuss et al. (2012) report data suggesting that participants can form expectations of where an event will occur on the basis of non-consciously presented cues. Duthoo et al. (2012) highlight the role of task repetition expectancy in task-switching by varying switch rate contingencies. Fröber and Dreisbach (2012) report data showing that positive affect with low arousal reduced proactive control as indicated by response cueing effects. Umbach et al. (2012) explored how explicit expectations feed into preparatory processes, over and above demand for preparation.

A final group of contributions targets temporal anticipation in the control of voluntary action. Predicting the temporal onset of an event by means of a warning cue allows for temporal orienting and anticipation of an upcoming event. In a brief review, Weinbach and Henik (2012) discuss whether the temporal orienting function of warning cues can be dissociated from cue-based increases of alertness. Finally, de la Rosa et al. (2012) show that temporal preparation guided by regular rhythms is not subject to working memory interference and facilitates performance irrespective of concurrent working memory load.
